# Mitochondria-Targeted Peptide SS31 Attenuates Renal Tubulointerstitial Injury via Inhibiting Mitochondrial Fission in Diabetic Mice

**DOI:** 10.1155/2019/2346580

**Published:** 2019-06-02

**Authors:** Shi-kun Yang, Ai-mei Li, Ya-chun Han, Can-hui Peng, Na Song, Ming Yang, Ming Zhan, Ling-feng Zeng, Pan-ai Song, Wei Zhang, Shi-qi Tang, Hao Zhang

**Affiliations:** ^1^Department of Nephrology, the Third Xiangya Hospital, Central South University, Changsha, Hunan Province, China; ^2^Department of Clinical Laboratory, the Third Xiangya Hospital, Central South University, Changsha, Hunan Province, China; ^3^Institute of Kidney Disease, Central South University, Changsha, Hunan Province, China; ^4^International Medicine Department, Ningbo First Hospital, Zhejiang University, Ningbo, China

## Abstract

**Objective:**

Renal tubular injury is an early characteristic of diabetic nephropathy (DN) that is related to mitochondrial dysfunction. In this study, we explore the effects and mechanisms of mitochondria-targeted peptide SS31 on renal tubulointerstitial injury in DN.

**Method:**

40 C57BL/6 mice were randomly divided into control group, STZ group, STZ+SS31 group, and STZ+normal saline group. SS31 was intraperitoneally injected to the mice every other day for 24 weeks. Renal lesions and the expression of Drp1, Mfn1, Bcl-2, Bax, Caspase1, IL-1*β*, and FN were detected. In *in vitro* studies, HK-2 cells were incubated with different concentrations of D-glucose (5, 30 mM) or combined with SS31 and Drp1 inhibitor Midivi1. Mitochondrial ROS, membrane potential, and morphology have been detected to evaluate the mitochondrial function.

**Results:**

Compared with diabetic mice, the levels of serum creatinine and microalbuminuria were significantly decreased in the SS31 group. Renal tubulointerstitial fibrosis, oxidative stress, and apoptosis were observed in diabetic mice, while the pathological changes were reduced in the SS31-treatment group. SS31 could decrease the expression of Drp1, Bax, Caspase1, IL-1*β*, and FN in the renal tissue of diabetic mice, while increasing the expression of Mfn1. Additionally, mitochondria exhibit focal enlargement and crista swelling in renal tubular cells of diabetic mice, while SS31 treatment could partially block these changes. An *in vitro* study showed that pretreatment with SS31 or Drp1 inhibitor Mdivi1 could restore the level of mitochondrial ROS, the membrane potential levels, and the expressions of Drp1, Bax, Caspase1, IL-1*β*, and FN in HK-2 cells under high-glucose conditions.

**Conclusion:**

SS31 protected renal tubulointerstitial injury in diabetic mice through a decrease in mitochondrial fragmentation via suppressing the expression of Drp1 and increasing the expression of Mfn1.

## 1. Introduction

Diabetic nephropathy (DN) is a severe complication of diabetic patients [1, 2]; it not only decreases the quality of life in DN patients but also brings serious economic burden on society. Unfortunately, the pathogenesis of DN is still not fully understood. Conventional wisdom suggested that glomerular lesions played the major roles in the progression of DN, while tubulointerstitial injury was regarded as a secondary lesion. But recent studies have shown that tubulointerstitial injury could serve as a primary pivotal site for the development of DN [3, 4]. As renal tubular reabsorption required a large amount of adenosine triphosphate (ATP) from mitochondria, it indicated that normal mitochondrial function was of greatest importance for maintaining a good functionality kidney. The application of mitochondrial dysfunction in DN tubulointerstitial damage is now receiving more and more recognition [5], and potential new pharmacological therapy that targeted on mitochondria may be effective in fighting DN.

Recently, a novel antioxidative peptide targeted on mitochondria named MTP131 or SS31 was designed [6]. SS31 peptide (H-D-Arg-Dmt-Lys-Phe-NH2) could specially concentrate in the inner mitochondrial membrane. It has been demonstrated that SS31 has excellent therapeutic efficacy in myocardial injury, neurodegeneration injury, and diabetic complications [7–10]. Our previous study demonstrated that mitochondria-targeted peptides (MTP131 and SPI20) could prevent contrast-induced acute kidney injury in rats [11]. In addition, *in vitro* experiments showed that SS31 could attenuate hypoxia-induced renal tubular epithelial cell apoptosis [12]. Furthermore, Hou et al. found that SS31 attenuated renal injury via decreasing mitochondrial ROS in diabetic mice [13]. However, the protective effect of these peptides on diabetes-induced renal tubulointerstitial injury was incompletely understood. Therefore, we performed this study to explore the effects and mechanisms of SS31 on DN both in vivo and in vitro.

## 2. Research Design and Methods

### 2.1. Cell Lines and Reagents

Human proximal tubular epithelial cells (HK-2 cells) were cryopreserved at the Institute of Kidney Disease, Central South University. SS31 was synthesised and provided by Chinapeptide Co. Ltd. (Shanghai, China). Streptozocin (STZ) was obtained from Sigma-Aldrich (USA). The selective Drp1 inhibitor Mdivi1 (ab144589) was obtained from Abcam (UK). Anti-fibronectin (FN) antibody (sc-52331), anti-Bcl-2 antibody (sc-56015), anti-IL-1*β* antibody (sc-52012), and anti-Bax antibody (sc-20067) were obtained from Santa Cruz Biotechnology (Santa Cruz, CA). Anti-Drp1 rabbit monoclonal antibody (ab184247), anti-Mfn1 mice monoclonal antibody (ab57602), and Caspase1 antibody (ab138483) were purchased from Abcam (UK). The TUNEL assay kit (ab66110) and anti-*β*-actin antibody (ab8226) were purchased from Abcam (UK). Secondary antibodies in this study were purchased from KangChen Bio-tech (Shanghai, China). Other materials, including bovine serum albumin and low-glucose DMEM medium, were purchased from Gibco (USA).

### 2.2. Animal Experimental Design

A total of 40 eight-week-old C57BL/6 mice (about 20 g body weight) were purchased from Slyke Jingda Biotechnology Company (Hunan, China), then they were divided into 4 groups. The control group was injected with sodium citrate buffer only (*n* = 10). The second group was injected intraperitoneally with STZ (40 mg/kg body weight) for 5 consecutive days (*n* = 10), and mice with glucose levels > 16.7 mmol/l were considered a diabetic model. If the level of blood glucose did not meet the standard, the mice had to resume taking injection of STZ until reaching blood glucose levels > 16.7 mmol/l. The third group of STZ-induced diabetic mice was injected with normal saline (NS) (5 ml/kg) (*n* = 10). The fourth group of diabetic mice was intraperitoneally injected with SS31 (3 mg/kg body weight) every other day for 24 weeks. They were killed at 24 weeks following the onset of STZ-induced diabetes. The sera and kidneys were harvested for further detection. The animal experiments were approved by the Ethics Review Committee of the Third Xiangya Hospital, Central South University.

### 2.3. Morphological Studies

Renal tissue sections were cut for hematoxylin-eosin (H&E), periodic acid-Schiff (PAS), and Masson's staining as described previously; glomerular and tubular injury was analyzed using a semiquantitative scoring system as previously described [14].

### 2.4. Assessment of Biochemical Index

Blood glucose was tested using a blood glucose monitor (Roche Accu-Chek, Germany) every two weeks. Mice were placed in individual metabolic cages for a 24-hour urine collection. A mouse urine albumin ELISA kit (Bethyl Laboratories, USA) was used to measure urine albumin concentrations. Serum creatinine, triglyceride, and cholesterol levels were measured by an automated biochemical analyzer (Hitachi 7600, Japan).

### 2.5. Renal Tissue Immunohistochemistry (IHC) and Apoptosis Assessment

Mouse renal tissue sections (3 *μ*m thick) was prepared; after deparaffinization, rehydration, and antigen retrieval, the sections were incubated with various primary antibodies FN (1: 100 dilution), Bcl-2 (1:100 dilution), Bax (1:100 dilution), IL-1*β* (1:100 dilution), Caspase1 (1:100 dilution), Mfn1 (1:100 dilution), and Drp1 (1:100 dilution) and then incubated with secondary antibodies; the sections were finally prepared for DAB reaction. Renal cell apoptosis assessment was performed using TUNEL staining as previously described [15].

### 2.6. Cell Culture and Treatment

HK-2 cells were maintained in media containing 5-30 mM D-glucose and other interventions: HK-2 cells maintained in 5 mM D-glucose (LG), HK-2 cells maintained in 30 mM D-glucose (HG), HK-2 cells treated with HG plus SS31 (100 nM), HK-2 cells treated with HG plus Mdivi1 (50 *μ*M), and HK-2 cells maintained in HG medium and normal saline. HK-2 cells treated with various agents were incubated for the indicated time (72 h).

### 2.7. Western Blotting Studies

Briefly, after the fractionated proteins were transferred onto a nitrocellulose membrane, they were incubated with various primary antibodies anti-FN (1:1,000), anti-Bcl-2 (1:1,000), anti-Bax (1:1,000), anti-IL-1*β* (1:1000), anti-Caspase1 (1:1,000), anti-Mfn1 (1:1,000), anti-Drp1 (1:1,000), and anti-*β*-actin (1:1,000); the membranes were then immersed in solutions containing secondary antibodies. The ECL system (Amersham, USA) was used for autoradiograms [16].

### 2.8. Cell Immunofluorescence

After HK-2 cells were treated with various agents, the cells were first immersed in MitoTracker Red (1:1000) solution, then the cells were fixed and permeabilized. After that, the cells were incubated with primary antibody (Drp1, 1:100 dilution). The cells were then incubated with FITC-conjugated secondary antibody and DAPI, and finally a confocal laser scanning microscope was used to capture images (Zeiss LSM 780).

### 2.9. Confocal Microscopy

A LSM 780 META laser scanning microscope (Zeiss LSM780) was used to complete the confocal microscopy examination. The LSM 510 software (Zeiss) was used for image analysis [17].

### 2.10. Examination of Mitochondrial Morphology Using Electron Microscopy

We used transmission electron microscopy (EM) to observe mitochondrial morphology. Briefly, renal cortices were minced into 1 mm^3^ pieces, then renal cortices were fixed with 2.5% glutaraldehyde; lastly, thin sections were prepared for EM to delineate the mitochondrial morphology in tubules.

### 2.11. Assessment of Mitochondrial ROS and Mitochondrial Membrane Voltage Potential (MMP, ΔΨm)

HK-2 cells were incubated with MitoSOX and examined by confocal microscopy to assess mitochondrial ROS levels. The cells were stained with TMRE and examined by confocal microscopy to evaluate the perturbations of MMP (ΔΨm). Mean fluorescence intensity was calculated based on 10 randomly selected fields.

### 2.12. Measurement of Oxidative and Antioxidative Biomarkers

MDA content as well as SOD and GSH-PX activities were determined by chemiluminescence methods as previously described [11].

### 2.13. Statistical Analysis

We used SPSS 16.0 software to analyze the experimental results. The results were expressed as mean ± standard deviation (SD). To assess for the results of between-group differences, analysis of variance with post hoc Tukey test was used. *P* < 0.05 was considered statistically significant.

## 3. Results

### 3.1. Effects of SS31 on Biochemical Parameters in Diabetic Mice

**Figure 1:**
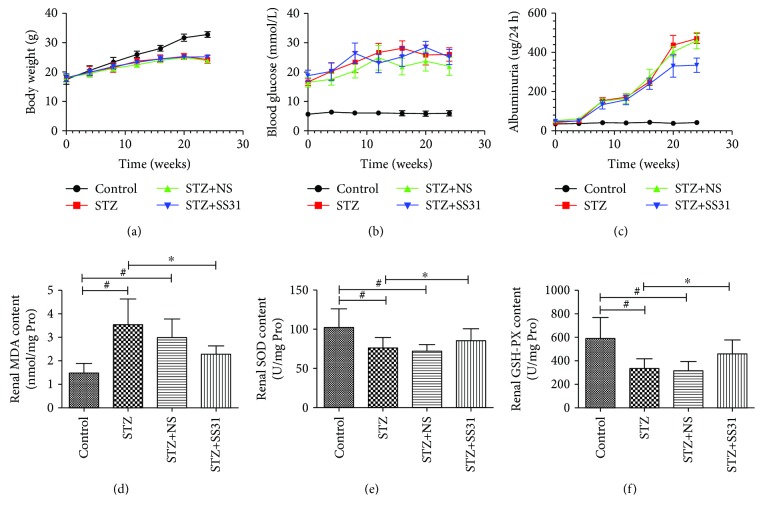
Effects of SS31 on biochemical index in diabetic mice. (a–c) Body weight, blood glucose, and proteinuria levels in mice from 0 to 24 weeks. (d) Renal malondialdehyde (MDA) concentrations of various groups. (e) Renal superoxide dismutase (SOD) concentrations of various groups. (f) Renal glutathione peroxidase (GSH-PX) concentrations of various groups. Data are presented as mean ± SD, ∗*P* < 0.01 vs. STZ groups, ^#^*P* < 0.01 vs. control groups. *n* = 10.

At the end of 24 weeks, 3 mice in the STZ group died, 3 mice in the STZ+SS31 group died, and 2 mice in the STZ+NS group died. Administration of SS31 for 24 weeks had no effect on body weight and blood glucose levels (Table 1, Figures 1(a) and 1(b)), while it decreased the level of proteinuria in STZ mice (Table 1, Figure 1(c)). Similarly, the levels of serum creatinine (Scr) and blood urea nitrogen (BUN) were increased in STZ mice, and SS31 treatment could restore these changes (Table 1). In addition, renal malondialdehyde (MDA) level was increased, while renal superoxide dismutase (SOD) and glutathione peroxidase (GSH-PX) levels were significantly decreased in diabetic mice; these changes were significantly reversed by SS31 treatment (Figures 1(d)–1(f)).

### 3.2. Effects of SS31 on Glomerular Injury and Tubulointerstitial Damage in Diabetic Mice

**Figure 2:**
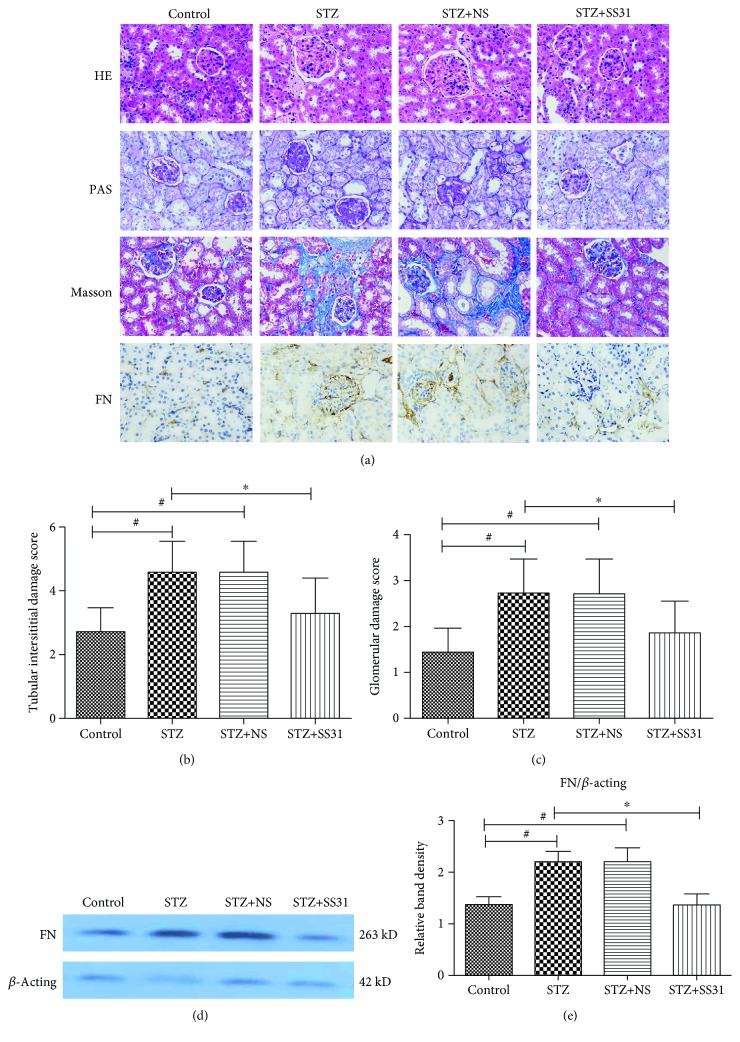
Effects of SS31 on renal tubulointerstitial damage in diabetic mice. (a) Renal tissue stained with HE, PAS, Masson trichrome, and immunohistochemical analysis of fibronectin (FN) (magnification ×400). (b) Tubulointerstitial damage scores, ∗*P* < 0.01 vs. STZ groups, ^#^*P* < 0.01 vs. control groups, *n* = 3. (c) Glomerular damage scores, ∗*P* < 0.01 vs. STZ groups, and ^#^*P* < 0.01 vs. control groups, *n* = 3. (d) Western blot analysis of FN protein. (e) Each bar graph represents the ratios of FN to *β*-actin, ∗*P* < 0.01 vs. STZ groups, ^#^*P* < 0.01 vs. control groups, *n* = 3.

It showed that SS31 treatment significantly alleviated mesangial matrix proliferation compared with untreated diabetic mice as indicated by HE, PAS staining, and glomerular damage scores (Figures 2(a) and 2(c)). In addition, increased renal interstitial fibrosis and tubulointerstitial matrix deposition were observed in the kidney of STZ-induced diabetic mice at the end of 24 weeks (Figures 2(a) and 2(b)). Furthermore, the expression of FN was significantly increased in the renal tubular interstitial region of STZ induced diabetic mice (Figures 2(a), 2(d), and 2(e)), while SS31 administration could markedly decrease these tubulointerstitial lesions (Figures 2(a), 2(d), and 2(e)).

### 3.3. Effects of SS31 on Renal Apoptosis in Diabetic Mice

**Figure 3:**
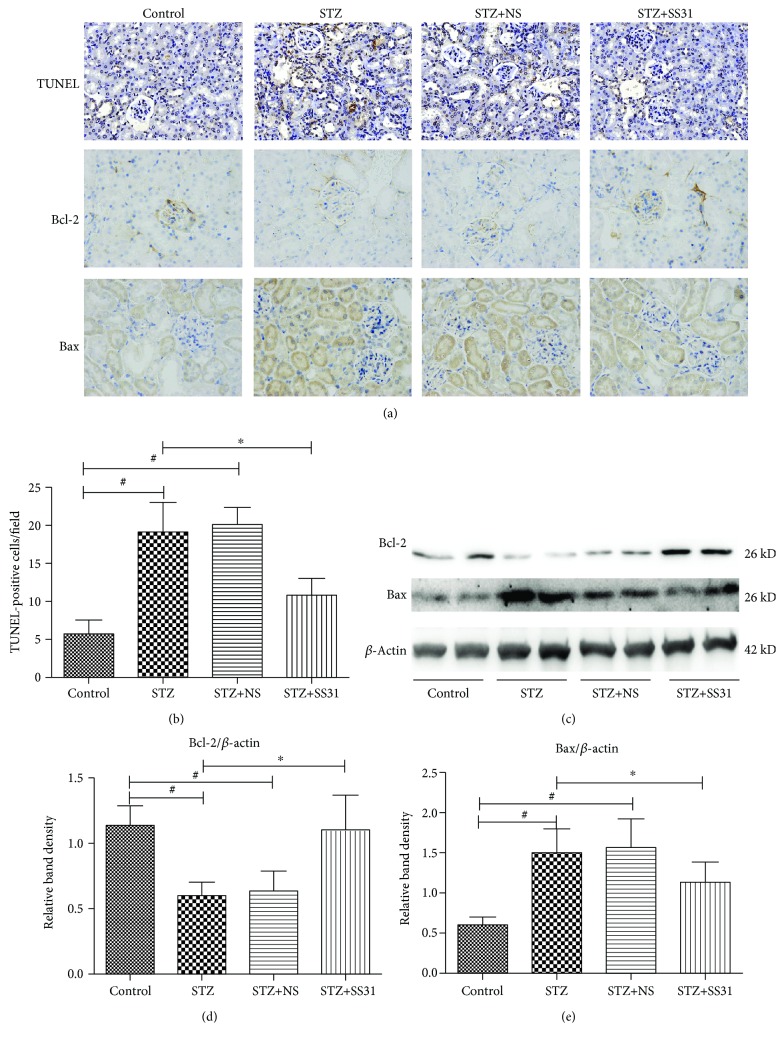
Effects of SS31 on apoptosis in renal tissue of diabetic mice. (a) TUNEL-IHC staining (upper panels) and immunohistochemical analysis of Bcl-2 (middle panels) and Bax (lower panels) in mouse renal tissue in various groups (magnification ×400). (b) Bar graphs represent quantification of tissues stained with TUNEL, ∗*P* < 0.01, vs. STZ groups, ^#^*P* < 0.01, vs. control groups, *n* = 3. (c) Western blot analysis of Bcl-2 (upper panel) and Bax (middle panel) protein expression. (d and e) Each bar graph represents the densitometric analyses of Bcl-2 to *β*-actin (d) and Bax to *β*-actin (e). ∗*P* < 0.01 vs. STZ groups, ^#^*P* < 0.01 vs. control groups, *n* = 3.

As shown in Figure 3, TUNEL-IHC staining showed that tubular epithelial cell apoptosis was observed in the kidney of STZ-induced diabetic mice, which was notably alleviated following SS31 treatment (Figures 3(a) and 3(b)). Furthermore, immunohistochemistry and Western blotting analysis showed that the expression of Bax in renal tissue from the STZ group was increased compared with that from the control group. Conversely, the expression of Bcl-2 was significantly decreased in the STZ group. SS31 treatment significantly increased the expression of Bcl-2 and decreased the expression of Bax protein in diabetic mice, respectively (Figures 3(a) and 3(c)–3(e)).

### 3.4. Effect of SS31 on the Expression of IL-1*β*, Caspase1, Mfn1, and Drp1 in Diabetic Kidneys

**Figure 4:**
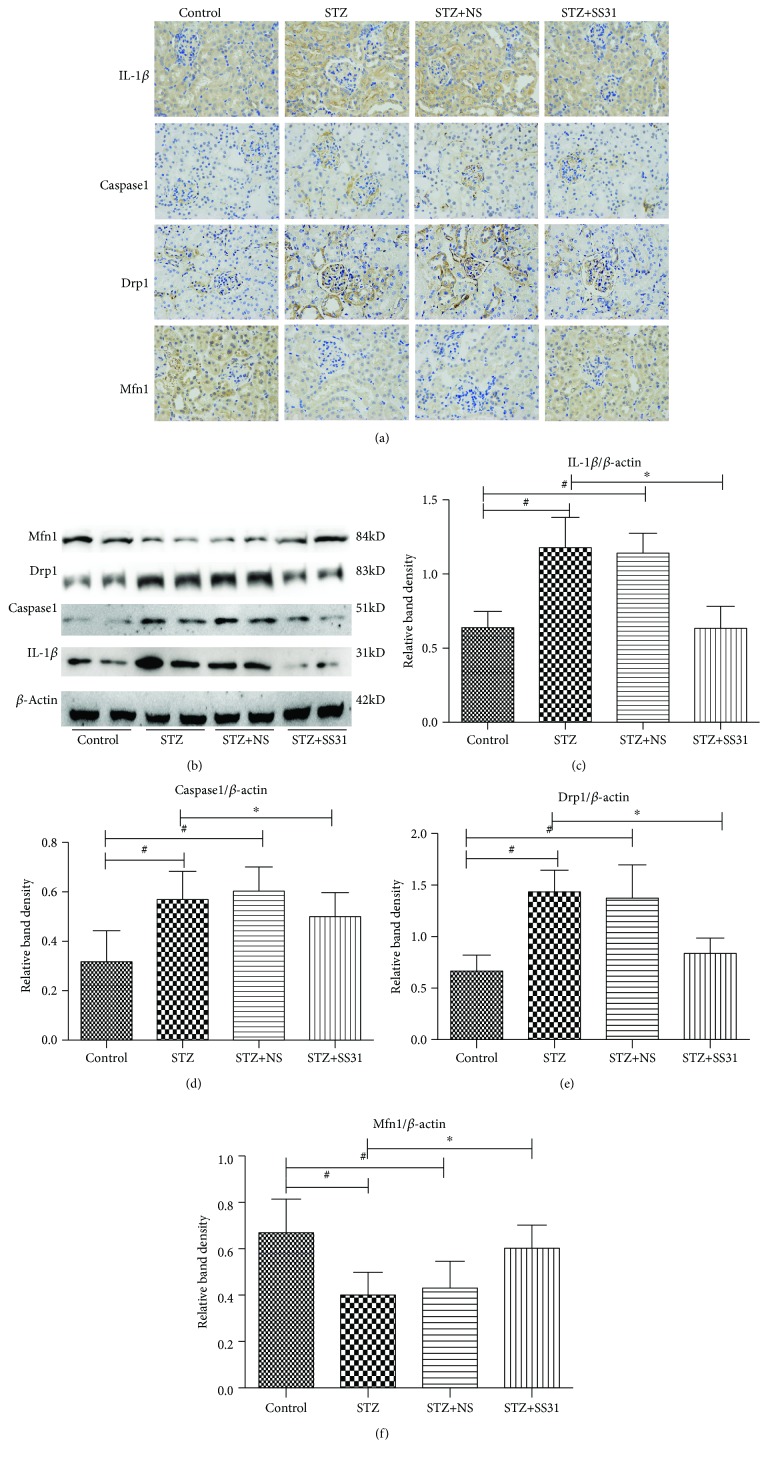
Renal IL-1*β*, Caspase1, Mfn1, and Drp1 expression in diabetic mice following SS31 treatment. (a) Renal immunohistochemical staining with anti-IL-1*β* antibody (upper panel), anti-Caspase1 antibody (middle panel), anti-Drp1 antibody (middle panel), and anti-Mfn1 (lower panel) (magnification ×400). (b) Western blot analysis of Mfn1 (upper panel), Drp1 (middle panel), Caspase1 (middle panel), and IL-1*β* (bottom panel) protein expression. (c–f) Densitometric analyses of the Western blotting results: IL-1*β* to *β*-actin (c), Caspase1 to *β*-actin (d), Drp1 to *β*-actin (e), and Mfn1 to *β*-actin (f). Values are mean ± SD, ∗*P* < 0.01, vs. STZ groups, ^#^*P* < 0.01 vs. control groups, *n* = 3.

IHC staining showed that renal IL-1*β*, Caspase1, and Drp1 expression was notably increased in diabetic mice; conversely, the expression of Mfn1 was decreased in STZ mice (Figure 4(a)). In addition, we found that Drp1 and Mfn1 were mainly expressed in renal tubules; however, after SS31 treatment for 24 weeks, these changes were significantly reversed. To confirm the above results, Western blot analysis was performed; similar results were observed regarding IL-1*β*, Caspase1, Mfn1, and Drp1 protein expression (Figures 4(b)–4(f)).

### 3.5. SS31 Restored Mitochondrial Morphology and Mitigated Mitochondrial ROS Generation

**Figure 5:**
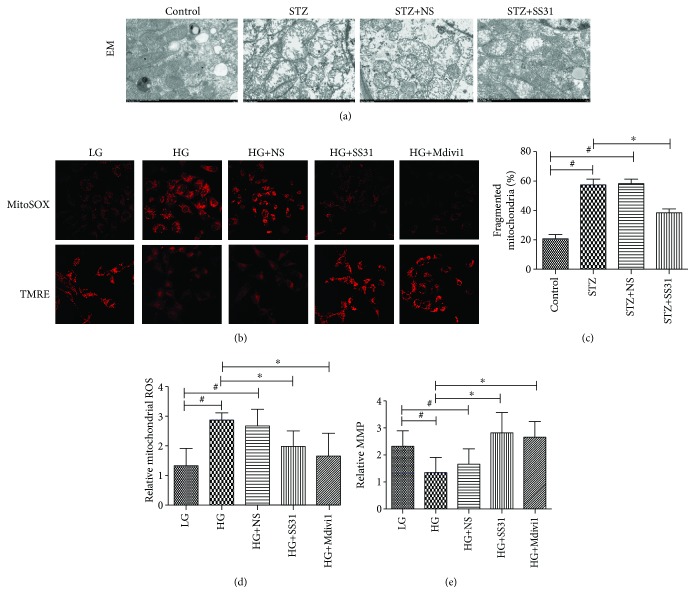
Effects of SS31 on mitochondrial morphology in the kidney of diabetic mice and mitochondrial ROS and mitochondrial membrane potential in HK-2 cells exposed to HG after SS31 administration. (a) EM analysis showed that the diabetic mouse renal tissues displayed obvious mitochondrial morphological changes; these changes were reversed by SS31 treatment (magnification ×5,000). (b) Representations of mitochondrial ROS levels (upper panel) and mitochondrial membrane potential (MMP, bottom panel) in HK-2 cells exposed to HG treatment with SS31 or Mdivi1 pretreatment (magnification ×400). (c) Relative percentages of fragmented mitochondria in the four groups. ∗*P* < 0.01 vs. STZ groups, ^#^*P* < 0.01 vs. control groups, *n* = 3. (d, e) Quantification of mitochondrial ROS production as measured with MitoSox Red staining (d) and MMP as measured with TMRE staining (e). ∗*P* < 0.01 vs. HG groups, ^#^*P* < 0.01 vs. LG groups, *n* = 3.

As shown in Figure 5, electron microscopy (EM) observation showed that the tubular mitochondria exhibit deformations in diabetic mice, such as mitochondrial crista swelling and focal disruption of the inner mitochondrial membranes (Figure 5(a)); SS31 treatment could obviously reverse these changes (Figures 5(a) and 5(c)). In vitro studies showed that HK-2 cells under an HG environment reduced mitochondrial membrane potential (MMP) and increased mitochondrial ROS levels, as indicated by TMRE and MitoSOX Red staining, respectively (Figures 5(b), 5(d), and 5(e)), these changes were reversed in cells pretreated with SS31. Interestingly, pretreatment with Drp1 inhibitor Mdivi1 also decreased the level of mitochondrial ROS in HK-2 cells exposed to an HG environment (Figures 5(b), 5(d), and 5(e)), and the MMP level was restored in HK-2 cells exposed to HG condition pretreatment with Mdivi1 (Figures 5(b), 5(d), and 5(e)).

### 3.6. SS31 Downregulated Drp1, Caspase1, and IL-1*β* Expression in HK-2 Cells Exposed to HG Conditions

**Figure 6:**
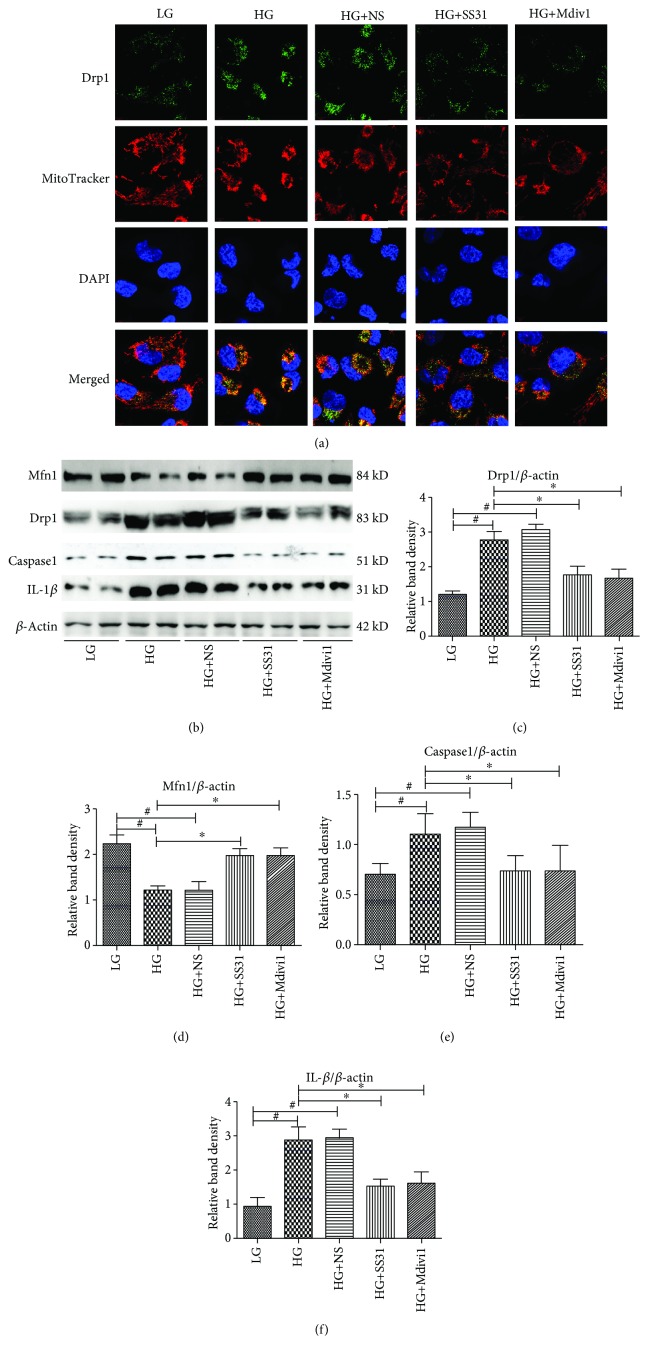
Effects of SS31 on Drp1, Mfn1, Caspase1, and IL-1*β* protein expression in HK-2 cells exposed to HG. (a) IF analysis of Drp1 expression and mitochondrial morphology in HK-2 cells exposed to HG conditions and pretreated with SS31 or Mdivi1. (b) Western blot analysis of Mfn1 (upper panel), Drp1, Caspase1 (middle panel), and IL-1*β* (bottom panel) protein expression in HK-2 cells exposed to HG conditions and pretreated with SS31 or Mdivi1. (c–e) Densitometric analyses of the Western blotting results: Drp1 to *β*-actin (c), Mfn1 to *β*-actin (d), Caspase1 to *β*-actin (e), and IL-1*β* to *β*-actin (f). The data are presented as mean ± SD, ∗*P* < 0.01 vs. HG groups, ^#^*P* < 0.01 vs. LG groups, *n* = 3.

Immunofluorescence studies indicated that HG increased Drp1 expression; additionally, MitoTracker staining showed increased mitochondrial fragmentation in HK-2 cells exposed to HG conditions (Figure 6(a)). These effects were reversed by SS31 treatment. In addition, we also found that pretreatment with Mdivi1 could decrease Drp1 expression in HK-2 cells under HG concentration (Figure 6(a)), then we investigated the effects of SS31 on Drp1, Mfn1, Caspase1, and IL-1*β* protein expression using Western blot analysis; as shown in Figure 6(b), increased expression of Drp1, Caspase1, and IL-1*β* were found in HK-2 cells exposed to HG condition, while the expression of Mfn1 was decreased in HK-2 cells under HG ambience. In addition, Western blot analysis showed that SS31 or Mdivi1 treatment could decrease the expression of Drp1, Caspase1, and IL-1*β* induced by HG; conversely, SS31 treatment could increase the expression of Mfn1 (Figures 6(b)–6(f)).

### 3.7. Discussion

The present study indicates that SS31 ameliorates renal tubulointerstitial injury in diabetic mice, which might be due to an antioxidant action, as well as decreasing mitochondrial fragmentation then restoration of mitochondria morphology via suppressing the expression of Drp1 and increasing the expression of Mfn1 in renal tubular epithelial cells.

The mitochondria target peptides included SS01, SS02, SS20, and SS31; the structural motif of these peptides was an alternation of aromatic residues and basic amino acids. It has been shown that SS31 could concentrate more than 1000-fold in the mitochondria [6]. The structure of these peptides includes tyrosine-containing analogs; they could scavenge free radicals (e.g., H_2_O_2_ and ONOO^−^). It was reported that SS31 might be beneficial for diseases associated with oxidative stress [18–20]. In addition, in vitro studies also demonstrated that these peptides could significantly attenuate the mitochondrial permeability transition (MPTP), cytochrome-C release, and mitochondrial swelling [21]. Our study found that long-term treatment with SS31 in diabetic mice could reduce renal oxidative stress levels, and more importantly, we found that SS31 treatment might alleviate renal tubulointerstitial injury induced by high glucose via regulating mitochondrial fragmentation for the first time.

Conventional wisdom suggested that glomerular injury was the major source of DN; however, recent studies indicated that tubulointerstitial lesions also closely correlated with the progression of DN, and the tubulointerstitial injury has been described as diabetic tubulopathy [16, 22, 23]. It has been found that renal tubular damage markers appeared before microalbuminuria; it indicated that tubular injury contributed to the primary renal injury in the pathogenesis of DN [24, 25]. In this study, notable changes including apoptosis and fibrosis in the tubulointerstitial were also observed in 24-week STZ-induced diabetic mice. The mechanisms of renal tubulointerstitial injury were not fully clear; previous research has shown that mitochondrial dysfunction played a crucial role in this process [15, 26]. This raised an interesting question of whether alleviation of mitochondrial dysfunction by exogenous therapeutic agents could delay the progression of DN. Our findings indicated that SS31, a mitochondrial ROS inhibitor, not only ameliorated morphological mitochondrial abnormalities but also reduced diabetic tubular injury.

The kidney is an organ needing continuous energy consumption due to the excretion and reabsorption process that existed in the renal tubule; there was a large amount of mitochondria both in the tubular and glomerular cells, particularly in the proximal tubular cells [5], and, notably, normal mitochondrial function was very critical for kidney cells. However, increasing *in vivo* and *in vitro* studies indicated that mitochondrial dysfunction played a critical biological role in the progression of various kidney diseases, including DN [5, 27], such as mitochondrial dynamic disorders and elevated mitochondrial oxidative stress. For instance, Yiu et al. found that the oxidative markers were significantly increased in the kidney of diabetic mouse, and reduced ROS generation could attenuate renal fibrosis [28]. In line with these observations, this research also confirmed that excessive mitochondrial ROS in the renal tissue of STZ induced diabetic mice.

Mitochondria are a class of highly shape-changed organelles which constantly undergo fusion and fission. In physiological conditions, they were elongated and filamentous, but the shape changed to fragment under stress including various kidney diseases [29–31]. Some key factors including fission mediators (Fis1, Drp1, and Dnm1) and the fusion proteins (Mfn1, Mfn2, and OPA1) controlled the balance of mitochondrial fusion and fission to maintain mitochondrial homeostasis [31]. This balance was disrupted under intracellular or extracellular stresses; mitochondria were changed from an elongated network into short rod spheres; this process is called mitochondrial fragmentation [32]. It has been demonstrated that Drp1 is an important regulator of mitochondrial fragmentation in diabetic conditions [33]. Importantly, excessive mitochondrial fission was related to increased mitochondrial ROS production and cellular apoptosis [34–36]. These previous findings suggested that Drp1 was a key regulatory factor for mitochondrial fragmentation in the renal cells of DN, and Drp1 might be a novel therapeutic target for DN. In this study, EM observation showed that the tubular mitochondria were swelled, shorter, and disrupted in 24-week diabetic mice, while treatment with SS31 could restore renal tubular mitochondria to be elongated structures. In order to explore the protection mechanisms of SS31, we found that SS31 treatment in STZ-induced diabetic mice could significantly attenuate renal oxidative stress and apoptosis and reduce the expression of the mitochondrial fission factor, Drp1, while the mitochondrial fusion protein (Mfn1) was increased after SS31 treatment. To confirm these findings, we performed an *in vitro* study using HK-2 cells. Increased expression of Drp1 and excessive mitochondrial ROS has been observed in the HK-2 cells exposed to high glucose, while these changes were reversed by SS31 treatment. Interestingly, similar results were noted in the group pretreatment with Mdivi1. It indicated that SS31 has a similar effect with Mdivi1 on inhibiting the expression of Drp1. In addition, *in vitro* studies showed that SS31 treatment could increase the expression of Mfn1. These findings indicated that SS31 mediated renal protection effects most likely via inhibiting Drp1 and activating Mfn1.

Because of a variety of reasons, there still existed several drawbacks in this study; first, we just examined the effects of SS31 on oxidative stress and apoptosis, while other effects such as anti-inflammatory effect had not been evaluated. Second, as we discussed above, mitochondrial fusion and fission were regulated by several factors (e.g., Drp1, Mfn1, OPA1, Mfn2, and Fis1), but in this study, we just examined the inhibiting effect of SS31 on Drp1 and the increasing effect of SS31 on Mfn1. Third, in the vitro experiment, we found that SS31 could inhibit the expression of Drp1 in HK-2 cells under HG condition, and the inhibiting effect was similar with Mdivi1; however, the results were suggestive and not cause-and-effect. In the future study, we will further investigate the more detailed molecular mechanism about SS31 regulating mitochondrial dynamics.

## 4. Conclusion

In conclusion, our data showed that SS31 could protect renal tubulointerstitial injury and reduce ROS and apoptosis in diabetic mice, which might be due to the decrease in mitochondrial fragmentation via suppressing the expression of Drp1 and increasing the expression of Mfn1.

## Figures and Tables

**Table 1:** Effect of SS31 on blood glucose, renal function, total cholesterol, and triglyceride and weight of STZ-induced diabetic mice.

Group	*n*	BW (g)	KW (mg)	KW/BW (mg/g)	BS (mmol/l)	Scr (*μ*mol/l)	BUN (mmol/l)	Proteinuria (*μ*g/ml)	Urine volume (ml)	Proteinuria (ug/24h)
Control	10	31.86 ± 2.35	198.30 ± 6.78	6.26 ± 0.55	7.13 ± 1.10	11.99 ± 1.05	9.93 ± 1.48	16.00 ± 1.76	2.71 ± 0.47	43.09 ± 8.24
STZ	10^&^	24.74 ± 1.55∗	214.01 ± 10.26∗	8.66 ± 0.35∗	27.04 ± 3.23∗	16.82 ± 2.43∗	12.98 ± 2.14∗	28.41 ± 4.36∗	16.05 ± 3.32∗	444.63 ± 67.05∗
STZ+saline	10^&^	23.98 ± 1.52∗	223.37 ± 5.18∗	9.35 ± 0.71∗	25.24 ± 3.84∗	16.32 ± 1.13∗	12.14 ± 1.42∗	28.81 ± 2.57∗	15.88 ± 3.68∗	454.98 ± 97.83∗
STZ+SS-31	10^&^	25.50 ± 1.32∗	204.57 ± 6.50^##^	8.04 ± 0.46∗^,##^	25.41 ± 3.57∗	13.62 ± 1.48^#^	10.43 ± 1.23^##^	19.91 ± 2.32^#^	16.43 ± 2.51∗	326.71 ± 60.79^##^

Note: BS: blood glucose; BW: body weight; KW: kidney weight of the right kidney; Scr: serum creatinine; BUN: blood urea nitrogen; TC: total cholesterol; TG: triglyceride. ^&^3 mice in the STZ group died, 2 mice in the STZ+saline group died, and 3 mice in the STZ+SS31 group died during 24 weeks. ^∗^*P* < 0.01, vs. control group; ^∗∗^*P* < 0.05, vs. control group; ^#^*P* < 0.01, vs. STZ group; ^##^*P* < 0.05, vs. STZ group.

## Data Availability

The data used to support the findings of this study are available from the first author and corresponding author upon reasonable request.
